# The Role of Bile Acids in Cardiovascular Diseases: from Mechanisms to Clinical Implications

**DOI:** 10.14336/AD.2022.0817

**Published:** 2023-04-01

**Authors:** Shuwen Zhang, Junteng Zhou, Wenchao Wu, Ye Zhu, Xiaojing Liu

**Affiliations:** ^1^Laboratory of Cardiovascular Diseases, Regenerative Medicine Research Center, West China Hospital, Sichuan University, Chengdu, China.; ^2^Health Management Center, West China Hospital, Sichuan University, Chengdu, China.; ^3^Department of Cardiology, West China Hospital, Sichuan University, Chengdu, China.

**Keywords:** Bile acids, metabolism, cardiomyocytes, noncardiomyocytes, cardiovascular diseases

## Abstract

Bile acids (BAs), key regulators in the metabolic network, are not only involved in lipid digestion and absorption but also serve as potential therapeutic targets for metabolic disorders. Studies have shown that cardiac dysfunction is associated with abnormal BA metabolic pathways. As ligands for several nuclear receptors and membrane receptors, BAs systematically regulate the homeostasis of metabolism and participate in cardiovascular diseases (CVDs), such as myocardial infarction, diabetic cardiomyopathy, atherosclerosis, arrhythmia, and heart failure. However, the molecular mechanism by which BAs trigger CVDs remains controversial. Therefore, the regulation of BA signal transduction by modulating the synthesis and composition of BAs is an interesting and novel direction for potential therapies for CVDs. Here, we mainly summarized the metabolism of BAs and their role in cardiomyocytes and noncardiomyocytes in CVDs. Moreover, we comprehensively discussed the clinical prospects of BAs in CVDs and analyzed the clinical diagnostic and application value of BAs. The latest development prospects of BAs in the field of new drug development are also prospected. We aimed to elucidate the underlying mechanism of BAs treatment in CVDs, and the relationship between BAs and CVDs may provide new avenues for the prevention and treatment of these diseases.

## 1. Introduction

Cardio-metabolic disease (CMD) is a broad term describing cardiovascular disease (CVD) caused by systemic metabolic changes. Metabolic changes are mechanically involved in almost all forms of cardiovascular disease [1]. CMD is the world’s leading cause of death and encompasses cardiovascular diseases, diabetes, and chronic renal failure [2].

Bile acids (BAs) are mainly synthesized in the liver and are the final product of cholesterol catabolism. Their components are cholesterol derivatives. Since the last century, evidence has shown that pathologically elevated BA circulation in liver disease is harmful to the heart [3, 4]. In the past few years, BAs have been discovered as circulating metabolites that can act as metabolic regulators by binding to multiple BA receptors. In view of their unique biological features, BAs play an important role in regulating multiple metabolic pathways, such as glucose, lipids and amino acids, as well as maintaining homeostasis of gut microbiota metabolism [5]. BAs can be present in most organs, tissues and cells that express its receptors. They are implicated in a variety of metabolic diseases and have evolved from a simple bile component to a complex metabolic integrator according to the researchers' understanding [6, 7].

BAs have not only been shown to play a key role in mediating oxidative stress, reactive oxygen species (ROS), mitochondrial dysfunction, cell membrane disruption and cellular damage [8]; more importantly, BAs and their metabolism are closely related to CVDs and metabolic disorders and help maintain cardiovascular function and health. BAs have two forms of effect on cardiac function: direct and indirect. Direct action requires BAs to interact with muscle cells, affecting myocardial contraction and conduction. These effects may or may not be receptor dependent. The indirect effects include multiple metabolic pathways, such as cardiac function regulation, cholesterol level regulation, and plaque formation in atherosclerosis [9].

In this paper, we summarize the role of different BA synthesis pathways and several regulatory mechanisms on related cells in the development of CVDs, as well as their role in the development and treatment of CVDs and related metabolic diseases. In addition, we introduce the potential therapeutic effects of ursodeoxycholic acid (UDCA) and other BA derivatives.

## 2. Physiological Functions of Bile Acids

### 2.1 The synthesis and metabolism of bile acids

BAs are the product of cholesterol metabolism in the liver. BA metabolism is the main pathway for the human body to remove cholesterol, and BAs are also important substances because they affect lipid absorption. Based on their structure, BAs can be divided into primary and secondary BAs. BAs can be further classified into bound and free BAs according to whether they are combined with glycine or taurine [10].

Currently, the major BAs identified in humans include chenodeoxycholic acid (CDCA), cholic acid (CA), and a small amount of lithocholic acid (LCA). UDCA and muricholic acid (MCA) are primary BAs in rodents rather than in humans. According to the different groups in the R1/R2/R3/X position, the lipophilicity of BAs is different. BAs are formed in the liver through a complex process and finally stored in the gallbladder. The formation process includes multiple reaction steps involving at least 17 different enzymes [11].

There are two ways to synthesize BAs, namely, the classical pathway and the alternative pathway [12]. In the classical pathway, cholesterol is catalyzed by cholesterol-7α-hydroxylase (CYP7A1) to first generate 7α-hydroxycholesterol as an intermediate product, which is further catalyzed to generate CA and CDCA [13]. CYP7A1 is the rate-limiting enzyme of the entire pathway, which determines the amount of BA produced. This is the rate-limiting step in the synthesis of BAs. Under normal conditions, at least ¾ of BAs are produced through this pathway [14]. The alternative BA pathway is theoretically present in the mitochondria of all cells or tissues. In this pathway, cholesterol is first catalyzed by sterol-27-hydroxylase (CYP27A1) to generate the intermediate product 27-hydroxycholesterol, which is then hydrogenated by sterol-7α-hydroxylase (CYP7B1) to form CDCA. The alternative approach mainly produces CDCA. Sterol-8α-hydroxylase (CYP8B1) plays a role in the synthesis of CA, and it can determine the ratio of CDCA to CA, the two main Bas [15]. In addition to CA and CDCA, primary BAs in mice also produce MCAs and UDCA [16]. MCAs are generally not detectable in humans. The synthesis of the final product of BAs requires modification by microorganisms in the gut. The 24-position carboxyl group of primary BAs is combined with glycine (in humans) or taurine (in mice), converted into secondary BAs, and excreted into bile. Secondary BAs are generally stored in the gallbladder and transported to the duodenum when needed. The amphiphilic structure of BAs makes them useful in emulsifying and absorbing some lipids and fat-soluble vitamins [17].

The liver contains very few BAs, and approximately 95% of the BAs secreted by the bile ducts are reabsorbed by microorganisms in the gut. BAs are primarily absorbed in the distal ileum in conjugated form by apical sodium-dependent bile acid transporters (ASBTs), recirculated through the portal vein into the liver, and then secreted again. This process occurs in the human body six times a day and is called *enterohepatic circulation* [14]. BAs form a metabolic axis between the liver and gut microbiota, which contributes to BA metabolic disturbances and significant changes in the composition of the microbiota. Hence, BA metabolism can be used as a new therapeutic strategy for metabolic diseases [18].

**Figure 1.**
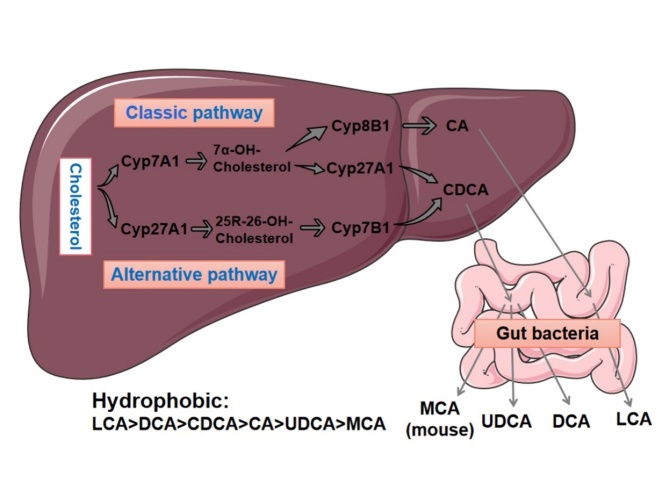
Synthesis and metabolism of BAs. There are two pathways to synthesize BAs, namely, the classical pathway and the alternative pathway[12]. The formation process includes multiple reaction steps. In the classical pathway, cholesterol is catalyzed by cholesterol-7α-hydroxylase (CYP7A1) to first generate 7α-hydroxycholesterol as an intermediate product, which is further catalyzed to generate CA and CDCA. The BA alternative pathway is theoretically present in the mitochondria of all cells or tissues. In this pathway, cholesterol is first catalyzed by sterol-27-hydroxylase (CYP27A1) to generate the intermediate product 27-hydroxycholesterol, which is then hydrogenated by sterol-7α-hydroxylase (CYP7B1) to form CDCA. The alternative approach mainly produces CDCA.

Deoxycholic acid (DCA), CDCA, LCA, and CA are crucial for the regulation of the BA pool. The cytotoxicity of BAs depends on their structure, but the hydrophobicity (lipophilicity) of BAs is related to the number and position of hydroxyl groups in their ring structure. The hydrophobicity-based order is as follows: LCA>DCA >CDCA>CA>UDCA>MCA. The most hydrophobic LCA is mostly excreted in the feces, with only a small amount being reabsorbed in enterohepatic circulation. The least toxic and most hydrophilic is UDCA. UDCA is synthesized in the gut by the dehydroxylation of free CDCA with the participation of bacteria. The hydroxyl group of UDCA is located in the β-ring, while CDCA is located in the α-ring. Interestingly, a growing body of research suggests that UDCA can play a protective role in CVDs (Fig. 1) [11].

According to previous studies, increased hydrophobic BA serum levels are associated with various metabolism-related diseases [16].

### 2.2 Bile acid receptors associated with cardiovascular diseases

BAs acting as signaling mediators are thought to bind to various receptors that affect the metabolism and regulation of lipid profiles [19]. These receptors include nuclear receptors, membrane receptors, and Ca^2+^-activated potassium (K+) (BK) channels [20-22].

These receptors have been recently discovered in endothelial cells, cardiomyocytes, vascular smooth muscle cells, and cardiac fibroblasts [23], indicating that BAs may have an impact on the cardiovascular system.

#### 2.2.1 Nuclear receptor

In the past few years, farnesoid X receptor (FXR)-mediated responses have been seen as critical for BA signaling [24]. More recently, other nuclear receptors, such as pregnane X receptor (PXR), liver X receptor (LXR), and vitamin D receptor (VDR), have also been found to play a role in the regulation of glucolipid metabolism [25].

FXR regulates the expression of CYP7A1 through a feedback mechanism by increasing BAs after food intake [19]. FXR serves as the primary target of most BAs. The hydrophobic side of the ligand-binding domain of FXR is able to bind to the hydrophilic side of BAs. CDCA is the most effective endogenous ligand for FXR binding compared with DCA, LCA, and CA. The functional study of FXR was first carried out in the intestine, where CDCA activated the expression of FXR and then mediated cholesterol secretion through intestinal acid-binding protein [22]. OCA is the most extensively studied FXR agonist and has been clinically evaluated [26]. Antagonists of FXR currently include UDCA and its conjugated form, glycine-UDCA [27, 28]. FXR activation is not only associated with maintaining normal cholesterol and triacylglycerol levels but is also expressed in the cardiovascular system. In addition, in the liver and gut, the activation of FXR affects regulators associated with CVD risks, such as glucolipid metabolism and endothelial function. FGF15 expression can be induced after intestinal FXR activation, which further improves glucose metabolism [29, 30]. However, there are studies showing the opposite results. Li et al. found that the improvement of glucose metabolism is achieved by inhibiting intestinal FXR signaling to alleviate the inhibition of L-cell glycolysis and GLP-1 secretion [31, 32]. Desai *et al* [33]. reported that the role of FXR in regulating BA levels is critical for organs such as the heart, and abnormally increased BA levels lead to cardiac dysfunction and cardiomyopathy in mice. However, when FXR activation is unrestricted, side effects such as pruritus, proatherosclerotic lipid profiles, and hepatotoxicity can also occur [27].

FXR, PXR, and VDR abolish BA-induced toxicity by downregulating the expression of cholesterol 7α hydroxylase, the rate-limiting enzyme in BA synthesis. An early animal study confirmed the significant role of PXR in lipid metabolism. Activation of PXR prevents high-fat diet- and obesity-induced insulin resistance by regulating energy and lipid metabolism [34].

Vitamin D (VD) deficiency may lead to bone and gastrointestinal-related diseases. Recently, CVDs, including heart failure and coronary heart disease, have been found to be associated with VD deficiency [35-38]. As a vital nuclear receptor that regulates calcium homeostasis, immunity, and cell differentiation, VDR is an endocrine nuclear receptor and is expressed in almost all tissues of the human body [39]. It has been reported that activation of VDR is able to participate in BA transport, metabolism, and detoxification by stimulating CYP3A. Furthermore, the natural ligand of LCA, 1α,25-dihydroxyvitamin D3 [1α,25(OH)2D3], can activate VDR. Both ligands activate the VDR signaling pathway via extracellular signal-regulated kinase 1/2, resulting in VDR phosphorylation and translocation into the nucleus. The selective binding of LCA acetate to VDR was 30-fold higher than that of LCA itself, but the specific binding to FXR and PXR was lower [40].

LXR plays a critical role in the metabolism, transport, and excretion of BAs and maintains cholesterol homeostasis [41]. Endogenous sterols and oxidized derivatives of cholesterol activate LXR. When intra-cellular oxytocin levels increase, activated LXRs are able to protect cells from high levels of cholesterol [42]. Unfortunately, the upregulation of the CYP7A1 gene and ATP-binding cassette caused by the cholesterol/LXR signaling pathway has not been observed in the human liver and has only been confirmed in animal models. Most current research focuses on other nuclear receptor pathways, and further exploration of the LXR activation pathway is needed.

#### 2.2.2 Membrane receptor

BAs also bind to three membrane G-protein-coupled receptors (the Takeda G-protein-coupled receptor 5 [TGR5], muscarinic [M] receptor, and S1P receptor), all of which are independent of nuclear hormone receptors and participate in cascades that activate intracellular effectors [40].

Approximately 10 years after the discovery of FXR, TGR5 was the first reported specific membrane receptor for Bas [43]. TGR5 is highly expressed not only in liver, adipose, and other tissues but also in the heart to some extent. TGR5 mRNA has been found in human, mouse, rabbit, and bovine cardiac tissue [44]. Moreover, different types of cells exhibit TGR5 expression, such as muscle, endocrine gland, and immune cells, as well as adipocytes [45].

Both LCA and DCA in secondary BAs are potent ligands for TGR5, and they can affect several important metabolic pathways, such as thermogenesis, glucose homeostasis, and energy metabolism. The activation of TGR5 signaling regulates several metabolic homeostasis pathways in the following ways: (1) improving insulin resistance by inducing type 2 iodase to increase energy expenditure in brown adipose tissue [46] and increase glucagon-like peptide-1 (GLP-1) secretion in enteroendocrine cells [47]; (2) reducing lipid load and inflammation in macrophages to prevent atherosclerosis [11], and (3) reducing blood vessel and liver damage to ameliorate nonalcoholic steatohepatitis [48]. In addition, the immunomodulatory function of TGR5 participates in various pathophysiological processes of multiple systems and exerts an inhibitory effect on inflammatory states [49], such as colitis [50, 51]. Steatohepatitis [52], atherosclerosis [53], sepsis [54], and inflammation related to type 2 diabetes [55].

The muscarinic (M) receptor is also a G-protein-coupled receptor (GPCR), and it is mainly expressed in the intestinal smooth muscle and gastrointestinal tract, including the five receptors M1-M5 [56]. The M2 receptor binds taurocholic acid and can affect transient calcium amplitudes by inhibiting cyclic AMP (cAMP), thereby reducing the contraction of cardiomyocytes [57]. The ligand of the M3 receptor is choline taurine.

Sphingosine-1-phosphate, another GPCR, has also been shown to be sensitive to BAs. As the most efficient substrate for sphingolipids, S1P is produced by sphingolipid kinase, catalyzed by sphingolipid phosphorylation. There are five subtypes of S1P receptors, namely, S1P1R, S1P2R, S1P3R, S1P4R, and S1P5R [58]. S1P1R, S1P2R, and S1P3R are mainly present in the heart, while S1P4R and S1P5R are only found in the immune and nervous systems [22]. S1P1R is the most important expressed isoform in cardiomyocytes, and its activation antagonizes adrenergic receptor-mediated contractility by inhibiting cAMP formation.

Secondary BAs activate S1P2R, affecting cell states by promoting apoptosis or survival signaling. Taurocholic acid promotes cholangiocarcinoma growth by inducing S1P2R expression [59]. S1P2R regulates liver glucose and lipid metabolism through the ERK1/2 and AKT signaling pathways [22]. Studies have shown that S1P2R and S1P3R protect against ischemia/reperfusion injury in mice. S1P agonists have a bradycardia effect, which can be mediated by low levels of S1P3R. S1PR is involved in various physiological activities of cardiac fibroblasts, such as proliferation, remodeling, and differentiation. S1PR-mediated pathways are also involved in hepatic fibrogenesis, regulating hepatic myofibroblast motility and vascular cell maturation and angiogenesis. Furthermore, in endothelial cells and smooth muscle cells, S1PR participates in endothelial cell responses and mediates peripheral vascular tone [60].

#### 2.2.3 BK_Ca_ channels

In addition to the known nuclear and membrane receptors, BAs have been proven to activate nonclassical receptor responses. Among these, the large-conductance calcium-dependent potassium channel (BK_Ca_) has the potential to increase the activity of BK_Ca_ in smooth muscle cells. Since this receptor mainly mediates ionic changes, it may play an indispensable role in related functions of cardiac conduction.

The activation of BK_Ca_ channels requires higher concentrations of BA than FXR or PXR. According to the findings of Bukiya *et al*., LCA can enhance the activity of BK_Ca_ channels in vascular myocytes [61]. The systemic vasodilation induced by BA in hepatobiliary diseases may be caused by the relaxation of VSMCs through the activation of BK_Ca_. In another study, the activation of BK_Ca_ channels by taurine-coupled hydrophobic BAs resulted in the outward expansion of potassium currents, shortened action potential duration, and negative inotropic effects. Additionally, BAs have been shown to increase the risk of cirrhotic cardiomyopathy by activating the BK pathway in cirrhotic patients [62].

Therefore, the contact of BAs with different receptors in different tissues may determine their function and level of regulation. The above studies suggest that BAs function as useful biomarkers in human CVDs. There are reports that the activation of these BA receptors may be dependent on the BA conversion activity of certain gut microbiota, providing a key clue linking cardiovascular diseases with microbiota composition and activity, which warrants further study. Table 1 summarizes the BA receptors and their expression in cardiovascular cells and tissues.

**Table 1:** Expression of BA receptors associated with cardiovascular function.

Receptor	Organ/Tissue	Cells type	Ligand	Ref.
FXR	Liver, gut, atherosclerotic blood vessels	Cardiomyocytes/Endothelial cells/Vascular smooth muscle cells	CA, CDCA, LCA, DCA	[22, 62, 63, 78, 82]
VDR	Liver	Cardiomyocytes	LCA	[38, 77]
PXR	Liver, mesenteric arteries	Cardiomyocytes	LCA	[39]
TGR5	Liver, glands, fat, muscle, immune, endocrine glands, enteric nervous system	Cardiomyocytes/Endothelial cells/Cardiac fibroblasts	CA, DCA, CDCA, LCA, TCDCA	[43, 81]
M	Nervous, intestinal, gastrointestinal	Cardiomyocytes/Endothelial cells	TC, LCT, TCA	[55, 71]
S1P	Liver, nervous, immune	Cardiac fibroblasts/Endothelial cells/Vascular smooth muscle cells	TCA, UDCA	[19, 58, 80]
BK^Ca^	Liver, brain	Cardiomyocytes/Vascular smooth muscle cells	LCA	[9, 59]

## 3. Bile Acid Metabolism in Cardiomyocytes and Noncardiomyocytes

### 3.1 Bile acid metabolism in cardiomyocytes

The heart is composed of noncardiomyocytes (70%) and cardiomyocytes (30%) [63]. BAs have direct and indirect effects on cardiac function. Their indirect effect is to directly affect the contraction and conduction of the myocardium through the interaction between BAs and cardiomyocytes.

In animal experiments, injecting large doses of BAs into animals causes significant bradycardia, indicating the cardiotoxicity of BAs and that BAs have time-varying and dose-dependent effects on cardiomyocytes [29].

In the heart, both cardiomyocytes and fibroblasts express FXR. FXR has a distinct stimulus-dependent effect in regulating cardiomyocyte injury [29]. Pu *et al*. [64] used cultured cardiomyocytes to prove that FXR expressed in cardiomyocytes was activated through mitochondrial death signaling. This finding was validated in a mouse model of myocardial ischemia/reperfusion injury *in vivo* [65]. In contrast, Xiaoli *et al.* found that FXR activation ameliorated cardiomyocyte damage induced by oxidative stress [66]. Furthermore, FXR activation reduced cardiomyocyte viability by triggering apoptosis. Therefore, it is speculated that FXR signaling is involved in several cardiac diseases associated with cardiomyocyte growth and apoptosis. FXR also regulates cardiac lipid accumulation in obese and diabetic patients by inducing the expression of β-oxidative genes in cardiomyocytes [67, 68]. As an agonist of FXR, GW4064 can significantly improve insulin resistance and cardiomyocyte disorders [69].

Clinical studies have demonstrated that within a certain concentration range, CDCA and DCA exert either a negative temporal effect by inhibiting the activity of the rat cardiac sinus node or a positive inotropic effect by increasing the concentration of Ca^2+^ in the cytoplasm of cardiomyocytes [70]. According to previous studies, LCA can reduce the apoptosis rate of cardiomyocytes [71].

The rate and pressure of myocardial contraction are determined by the rate of calcium influx. The t-tubule is the location that regulates intracellular calcium flow and contractility, and VDR is localized in the t-tubule of cardiomyocytes. Thus, loss of cardiomyocyte VDR selectivity results in cardiomyocyte hypertrophy, which affects the systolic and diastolic function of cardiomyocytes [72]. VD supplementation improves the left ventricular structure and restores cardiac function in patients with HF, further indicating that VDR is important in the maintenance of normal function in cardiomyocytes [73].

In addition, when the TGR5 gene is deleted in cardiomyocytes, the ability of the myocardium to adapt to the three stressors (physiological, inotropic, and hemodynamic stress) is significantly impaired. TGR5 can be readily targeted by BAs and their synthetic analogs and then regulate the expression of cardiac PDK4 by activating the Akt signaling pathway to improve cardiac glucose metabolism and play a beneficial role in patients with different types of CVDs [71].

Taurine cholic acid (TCA) acts as a partial agonist of M2 receptors. The binding of the M2 receptor to taurocholic acid inhibits cyclic AMP (cAMP), reduces myocardial cell contraction, and induces arrhythmia in CMs [74]. In cardiomyocytes, the activation of the S1P1 receptor, one of the most important expression subtypes of S1P, can also inhibit the synthesis of cAMP and antagonize adrenergic receptor-mediated contractility. Mohamed *et al*. reported that the protective effect of UDCA on CMs against hypoxia is partly similar to that of FTY720 (an S1P receptor agonist), which maintains normal intracellular [Ca^2+^] through S1P1 receptor-mediated hypoxia [75]. UDCA was also able to reverse fetal cardiomyocyte injury in a rat model of ICP [76]. Another study revealed that DCA and CDCA can also induce the production of cyclic adenosine monophosphate and reduce the contraction rate of neonatal mouse ventricular myocytes [77].

In summary, the relationship between BAs and cardiomyocytes involves the regulation of multiple receptors, which is a complex and multifactorial process.

### 3.2 Bile acid metabolism in endothelial cells

Endothelial dysfunction is one of the major drivers of CVDs such as atherosclerosis [78, 79]. FXR ligands in endothelial cells have been found to increase FXR expression, upregulate endothelial nitric oxide synthase (eNOS), reduce endothelin-1, and modulate angiotensin-II receptors, thereby inhibiting VSMC inflammation and migration [80, 81]. Endothelin-1 (ET-1) is the most effective vasoconstrictor currently available. GW4064, a chemical FXR agonist, was proven to increase eNOS expression [82]. Other studies have shown that activation of FXR prevents vasoconstriction mediated by increased eNOS and decreased ET-1 and that FXR can impair the vasorelaxation of endothelial cells under chronic stimulation [83, 84].

After identifying the role of FXR in lung endothelial cells, He *et al*. confirmed that CDCA activation resulted in a concentration-dependent decrease in endothelin-1 mRNA expression [22]. The expression of TGR5 was also found in aortic endothelial cells, which produce nitric oxide. S1P receptors present in endothelial cells can mediate endothelial cell responses to BAs. Furthermore, the BA-mediated activation of Ca^2+^-dependent K^+^ currents has been confirmed in endothelial cells [9]. Consistent with the effect of DCA on the receptors, the muscarinic M2 and M3 receptor responses to cardiac and vascular endothelial cells were attenuated in a model of liver cirrhosis [22].

### 3.3 Bile acid metabolism in vascular smooth muscle cells

Some data suggest that in vascular tissue, FXR not only regulates its own expression but also functions as a transcription factor in vascular smooth muscle cells (VSMCs). FXR regulates vasoconstriction and relaxation by altering the duration of other receptors in blood vessels and the production of active molecules.

In VSMCs, the expression of type II angiotensin receptors increases with FXR ligands. Studies have shown that FXR activation can inhibit the endothelin-1β-mediated induction of eNOS and COX-2 by upregulating ET-1 and eNOS, further inhibiting vascular smooth muscle cell inflammation and migration and finally inducing endothelial vasodilation [85]. However, chronic stimulation of FXR reduces cGMP sensitivity in smooth muscle cells and attenuates NO-dependent vasodilation [81]. Therefore, temporal variables should be considered when exploring BA receptor-related effects.

As another BA-sensitive receptor on VSMCs, S1PR2 mediates NO signaling and participates in peripheral vascular tone and endothelial cell responses. In addition, S1PR2 reduces NO levels in vascular injury by inhibiting the action of inducible NO synthase [86].

BAs also activate Ca^2+^-dependent K^+^ channels in VSMCs. In pressurized cerebral resistance arteries, blockers of BK_Ca_ channels are able to inhibit LCA-mediated endothelium-dependent vasodilation. In a mouse model, LCA was unable to stimulate arterial vasodilation after knockout of the BK β-1 subunit, indicating that the BK β-1 subunit plays a significant role in activating LCA [9]. Thus, these data suggest that BAs can stimulate vasodilation by activating BK_Ca_ channels in VSMCs and indicate a critical role for the BK β-1 subunit in CVDs.

### 3.4 Bile acid metabolism in cardiac fibroblasts

Cardiomyocytes and cardiac fibroblasts are the two most important resident cells in the heart, which participate in various pathophysiological processes of that organ and interact with each other.

The interleukin (IL)-1 family is considered essential in repairing and remodeling infarcted heart damage, and IL-1β is an important effector [87]. In an experiment involving the coculture of fibroblasts and cardiomyocytes, hypoxic stimulation showed that TGR5 mRNA expression was reduced in both types of cells. DCA inhibited the activation and expression of IL-1β in cardiomyocytes and fibroblasts under hypoxic conditions, and IL-1β mRNA expression was decreased in both cell lines [88]. Therefore, controlling BA metabolism by activating the DCA-TGR5 signaling pathway is thought to reduce postinfarction inflammation and improve cardiac function. This strategy may provide new therapeutic avenues for patients with myocardial infarction.

In the gut, FXR activation induces the expression of fibroblast growth Factor 19 (FGF19). In turn, FGF19 activates FGF receptor 4 (FGFR4) in the liver, which reduces BA synthesis by further inhibiting CYP7A1 [89]. Other studies have shown that S1PRs also play a role in the proliferation, remodeling, and differentiation of cardiac fibroblasts [19].

In summary, the relationship between BAs and CVD-related cells involves the regulation of multiple pathways and multisystem interactions.

**Table 2:** Behavior of bile acids and their derivatives via their associated receptors in cardiovascular disease.

Types of BA or its Derivatives	Receptors	Diseases	Roles in Diseases	REF.
UDCA	TGR5	Diabetic cardiomyopathy	Improvement of endoplasmic reticulum stress, blood glucose level and GLP-1 secretion in diabetic cardiomyopathy rats	[108]
	/	Atherosclerosis	Anti-atherosclerotic effects by reducing endoplasmic reticulum (ER) stress and pro-inflammatory responses	[122-124]
	M2/TGR5	Arrhythmia	Protection of the myocardium by antagonizing other hydrophobic BAs and cardiac wavelengths to mediate antiarrhythmic effects	[57, 130-135]
	TGR5	Heart failure	Enhancement of the adaptability of the heart to physiological, muscle strength, and hemodynamic stress	[137]
	FXR	Cirrhosis cardiomyopathy	Protection of liver cells by promoting bile flow, reducing liver enzyme levels and replacing hydrophobic BA	[10, 145]
DCA	TGR5	Myocardial infarction	Inhibition of inflammatory responses in cardiomyocytes and fibroblasts through activation of the DCA-TGR5 signaling pathway	[88, 98]
CA	PXR	Diabetic cardiomyopathy	Regulation of lipid and energy metabolism to combat high-fat diet-induced obesity and insulin resistance, and increase in insulin secretion in pancreatic B cells for antidiabetic effects	[102, 103]
OCA	FXR		Improvement of metabolic abnormalities and impaired glucose tolerance, including lowering blood glucose and insulin levels and reducing body weight and heart weight,and protection against diabetic cardiomyopathy by activating the FXR-mediated Nrf2 signaling pathway	[110, 111]
INT-747		Atherosclerosis	Downregulation of the vasoconstrictor endothelin-1, thereby preventing smooth muscle cell-mediated atherosclerotic effects and migration processes	[95]
			Inhibition of the accumulation of triglyceride- and phosphate-induced mineralization	[9]
INT-777	TGR5		Improvement of metabolic syndrome and atherosclerosis	[71]
GUDCA	FXR		1) Improvement of cholesterol homeostasis by modulating gut microbiota, and by inhibiting foam cell formation. 2) improvement of local chronic inflammation, lipid deposition, plaque area, and plaque stability to slow the progression of atherosclerosis	[120]
CDCA			Reduces hepatic lipolysis, cholesterol levels and bile acid efflux, activates hepatic FXR-BSEP signaling and reduces atherosclerotic damage	[119]
	LXR	Diabetic cardiomyopathy	Promotion of glucose metabolism by upregulating the expression of LXRs and increasing the secretion of GLP-1 and glucagon	[106, 107]

## 4. The Role of Bile Acid Metabolism in Cardio-vascular Diseases

Previous studies have shown that elevated concentrations of BAs can reduce the heart rate and cardiac contractility in rats. Taurine deoxycholic acid may improve cardiac contractility by inhibiting endoplasmic reticulum (ER) stress, apoptosis, inflammation, and fibrosis [90]. Additionally, elevated serum BA levels are related to adult arrhythmia, poor contractility of cardiomyocytes, and poor fetal outcomes in pregnant women with obstetric cholestasis [91]. In contrast, UDCA, the most hydrophilic BA, has been proven to help improve chronic heart failure and to play a protective role in cardiac ischemia-reperfusion injury and myocardial infarction [10].

**Figure 2.**
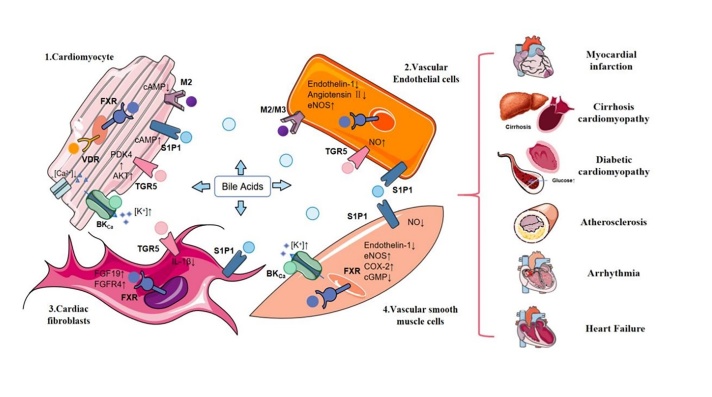
BAs affect cardiovascular disease by binding to various receptors that affect metabolism and regulation of lipid profiles. These receptors include nuclear receptors (FXR, PXR, VDR, LXR), G protein-coupled receptor (TGR5, muscarinic receptor, S1PR) and Ca^2+^-activated potassium (K+) (BK) channels. These receptors are highly expressed in cells associated with the cardiovascular system, such as cardiomyocytes, endothelial cells, cardiac fibroblasts and vascular smooth muscle cells. By binding to receptors on cells, BAs further influence intracellular regulators related to CVD risk. They are ultimately involved in the occurrence and development of cardiovascular system diseases such as cardiomyopathy, atherosclerosis, arrhythmia, and heart failure by affecting the relevant regulatory factors of cardiovascular disease risk.

Furthermore, the composition of BA pools has been altered in patients with chronic heart failure [92]. In patients with liver cirrhosis, cardiac dysfunction is closely related to the increase in serum BA concentrations [10]. Based on relevant studies, we know that different BAs may have different effects on cardiac function. Some recent evidence indicates that BAs not only affect the pathogenesis of metabolic diseases but may also serve as markers of these diseases [93]. Thus, BAs may be potential biomarkers of metabolic health and diseases. In clinical conditions, tracking BAs through advanced analytical techniques may provide a potential and effective avenue for identifying new treatments for cardiovascular diseases (Table 2, Fig. 2).

### 4.1 Bile acid metabolism in myocardial infarction

The pathogenesis of myocardial infarction (MI) and its complications involve a variety of metabolic disorders. Various inflammatory responses during heart remodeling after MI are critical for cardiac repair. Metabolic changes also affect systemic inflammatory activation status. Therefore, increased attention is directed to other pathways that modulate the inflammatory response by modulating metabolic pathways. There are an increasing number of studies on BA metabolites as signaling molecules that affect various cardiovascular functions [94]. In recent years, studies have suggested that BAs can directly regulate a variety of pathophysiological processes. Research has also confirmed that myocardial infarction is intrinsically linked to cholesterol metabolism regulated by Bas [95].

FXR agonists can improve cardiac dysfunction after myocardial infarction by stimulating adiponectin secretion [96]. Moreover, FXR knockout maintains cardiac function after myocardial infarction by reducing cellular fibrosis and chronic apoptosis [97]. DCA is one of the most potent activators of TGR5. The protective role of DCA in cardiac repair after myocardial infarction is based on its anti-inflammatory effect. The activation of DCA-TGR5 signaling can inhibit the inflammatory response of cardiomyocytes and fibroblasts, which helps DCA play a protective role in myocardial infarction [88, 98]. The levels of DCA in patients with AMI were significantly lower than those in controls. Interestingly, after DCA supplementation, the area of myocardial infarction was reduced, and heart function was also improved [99]. The authors of another study found that TGR5 regulates the function and subpopulation distribution of CD4^+^ T cells in the heart, thus playing a protective role in myocardial infarction [100]. UDCA and its conjugated metabolite GUDC have been reported to be decreased in patients with acute myocardial infarction [88].

In conclusion, DCA, as one of the strongest ligands of BAs, mainly mediates its biological function via TGR5, which plays a mitigating role in the process of myocardial infarction. Therefore, strategies aimed at regulating BA metabolism and related signal transduction to improve the inflammatory response may be helpful for patients with myocardial infarction.

### 4.2 Bile acid metabolism in diabetic cardiomyopathy

Diabetic cardiomyopathy (DCM) is considered the leading cause of high mortality from heart disease, and it is also one of the common cardiac complications in diabetic patients. Hyperglycemia and insulin resistance are key factors in the pathogenesis of DCM and are associated with inflammation, oxidative stress, and mitochondrial dysfunction. The pathological mechanisms underlying DCM include apoptosis, hyperglycemia and hyperlipidemia, the accumulation of extracellular matrix, the disturbance of calcium homeostasis in cardiomyocytes, and diastolic dysfunction [101].

In one animal study, researchers found that PXR activation mitigated obesity and insulin resistance caused by high-fat diets by regulating lipid and energy metabolism, indicating that PXR plays an important antidiabetic role [102]. Studies have shown that CA and CDCA are associated with a reduced risk of diabetes. CA exerts anti-diabetic effects by increasing insulin production in pancreatic B cells [103]. As an intestinal hormone, incretin GLP-1 stimulates insulin secretion and sensitivity, glucose production and lipolysis, and it also increases satiety, which is beneficial to the human body [104]. In previous studies, GLP-1 was shown to have cardioprotective effects, such as regulating cardio-myocyte function and reducing atherogenic plaque inflammation [105]. CDCA promotes glucose metabolism by upregulating the expression of LXRs and increasing the secretion of GLP-1 and glucagon [106, 107]. Additionally, GLP-1 secretion can be regulated by TGR5. In bariatric surgery, TGR5 improves glucose homeostasis [108].

Another study demonstrated that UDCA reduces ER stress in rats with diabetic cardiomyopathy, dissolves cholesterol formed in the gallbladder, and then reduces the absorption of cholesterol. Furthermore, UDCA has been shown to reduce oxidative damage induced by hydrophobic Bas [71]. A study by Basso *et al*. showed that an increase in UDCA and its conjugates increased insulin sensitivity during bariatric surgery [109]. Rat UDCA levels are elevated after partial gastrectomy, which correlates with the distribution of fat and the enhancement of insulin sensitivity. Ingestion of UDCA particles in healthy individuals improves blood glucose levels and GLP-1 secretion, promotes gastric emptying, and modulates glucose-induced insulin excretion [109]. Therefore, BAs may play a significant role in diabetic cardiomyopathy through TGR5-mediated GLP-1 secretion.

Because of the limited effect of UDCA, obeticholic acid (6-ethyl goose deoxycholic acid [OCA]) has been synthesized with CDCA. OCA is currently the most clinically advanced BA derivative. It is anticipated that OCA can be used as a new therapeutic drug to replace UDCA. OCA has a variety of biological and pharmacological applications. As a semisynthetic BA analog, it has a strong binding affinity to FXR [110]. Wu *et al.*[101]. confirmed that OCA improves metabolic abnormalities and reduces impaired glucose tolerance, including lowering blood glucose and insulin levels and reducing body weight and heart weight. Data have shown that in diabetic mice, OCA exhibits antioxidant activity and protects against diabetic cardiomyopathy by activating the FXR-mediated Nrf2 signaling pathway [111].

The metabolic effects of several drugs commonly used in the clinical treatment of diabetes are generally dependent on the regulation of BA metabolism. Among them, metformin is thought to have a hypoglycemic effect by reducing the intestinal absorption of BA [112]. The synthesis and metabolism of BAs may change with the course of diabetic cardiomyopathy. Therefore, more research is needed to determine whether the risk of diabetic cardiomyopathy can be reduced by interfering with the content of BA in the serum. These studies may provide new insights into the diagnosis and treatment of diabetic cardiomyopathy.

### 4.3 Bile acid metabolism in atherosclerosis

Several studies have shown that hydrophobic BAs can be blocked by inhibiting ER stress, free cholesterol-induced cell death in macrophages, and the presentation of major histocompatibility complex (MHC)-related antigens. Fat production increases the risk of developing athero-sclerosis and other cardiovascular diseases [113]. It has been reported that BA sequestrants regulate blood cholesterol levels, thereby affecting the formation of atherosclerotic plaques [114].

FXR has a tissue-specific role in the development and progression of atherosclerosis, and its prevention depends not only on hepatic FXR activation but also on global and intestinal depletion of FXR [115-117]. Activation of FXR is associated with regulators in the liver and gut that affect CVD, such as endothelial function, lipid and glucose homeostasis, and athero-sclerosis. It is associated with maintaining normal cholesterol triacylglycerol levels [19]. FXR also regulates inflammation in blood vessels. Synthetic FXR ligands were able to inhibit the inflammatory response of rat smooth muscle cells, which is induced by interleukin-1β, suggesting that FXR agonists have antiatherosclerotic potential [118]. Calcification is a feature of athero-sclerosis. The CDCA derivative INT-747 can inhibit the accumulation of triglyceride- and phosphate-induced mineralization. The anti-calcium effect of INT-747 is regulated by FXR, and when FXR is inhibited, the mineralization of CVCs is increased [9]. In ovariectomized mice, increased levels of CDCA in the liver activate hepatic FXR-BSEP (bile salt export pump) signaling and reduce atherosclerotic damage by reducing hepatic lipolysis, cholesterol levels, and bile acid efflux [119]. As a gut FXR antagonist, GUDCA may improve cholesterol homeostasis by modulating gut microbiota. In addition, by inhibiting foam cell formation, GUDCA improves local chronic inflammation, reduces lipid deposition and plaque area, and improves plaque stability to slow the progression of atherosclerosis [120].

OCA, another potent FXR agonist, has shown treatment efficacy in preventing high-fat diet-induced atherosclerosis. There are some data supporting the ability of FXR agonists to downregulate the vasoconstrictor endothelin-1, thereby preventing smooth muscle cell-mediated atherosclerotic effects and migration processes [95]. As shown, the role of FXR in atherosclerosis is complicated, and more research is needed to more fully evaluate the effects of long-term FXR stimulation on atherosclerosis, as well as more *in vivo* experiments to determine the BA-FXR interaction. LXR has also been reported to regulate CVDs such as atherosclerosis. Bradley *et al*. showed that activation of LXR reduces the formation of atherosclerotic lesions [10]. However, the BA/LXR signaling pathway mainly functions in animal models. Therefore, further research is needed on the BA/LXR signaling pathway.

One study claimed that in the heart, TGR5 can inhibit inflammation and the formation of atherosclerotic plaques, thereby improving atherosclerosis [121]. A semisynthetic derivative of CA, 6α-ethyl-23(S)-methylcholic acid (S-MECA, INT777), which acts as a TGR5 agonist, has been shown to improve metabolic syndrome and reduce atherosclerosis in mice. It is suggested that TGR5 plays a potential role in atherosclerosis prevention [71]. In addition, in bovine aortic endothelial cells, activation of TGR5 also inhibited NF-κB activity and induced NO production, inhibiting monocyte adhesion, macrophage lipid load and intraplaque inflammation and thereby preventing the accumulation of atherosclerotic plaque in the arteries [121].

Encouragingly, it has been reported that in a mouse model of diabetic atherosclerosis, hydrophilic BA-UDCA was able to exert antiatherosclerotic effects by alleviating ER stress and proinflammatory responses [122, 123]. An experimental study by Hanafi *et al.* reported that UDCA mediated the direct protection of the heart by regulating the ERK/Akt pathway [124].

Although BAs play a crucial role in the progression of atherosclerosis, the potential value of BA metabolism in the early stages of atherosclerosis remains unclear. Therefore, it is necessary to further explore the biological mechanism of BAs in the occurrence and progression of atherosclerosis.

### 4.4 Bile acid metabolism in arrhythmia

It has been gradually discovered and confirmed that high levels of BAs can cause various types of arrhythmias through various mechanisms. Furthermore, BA-induced arrhythmias are more common in fetuses than adults [125].

Data show that changes in the composition of BA pools in patients’ serum can induce atrial arrhythmias. In some *in vivo* experiments, the researchers found a significant increase in the proportion of BAs other than UDCA in the plasma of the atrial fibrillation group. Therefore, serum UDCA concentrations and the non-UDCA ratio may serve as independent predictors of atrial fibrillation [95]. Furthermore, the most hydrophilic BA, UDCA, has been proven to be cardioprotective against BA-induced arrhythmias in a cholestatic fetal heart model [126].

BA concentrations in patients with intrahepatic cholestasis (ICP) are associated with ventricular arrhythmias. Patients with PBC have a significantly prolonged corrected QT interval, which causes ventricular arrhythmias and further increases the risk of sudden death [127]. The increase in the concentration of BA in pregnant women with ICP could lead to the accumulation of BAs in fetal serum and fetal arrhythmia [128]. Sheikh *et al.*[129] stimulated the heart with the M2 receptor agonist carbachol and found the onset of bradycardia in mice. Moreover, abrogation of the M2 receptor improved TCA-induced cardiac arrhythmias in a fetal heart model. Therefore, it is suggested that TCA-induced arrhythmias are mediated by partial agonism of M2 receptors.

In another study, Ibrahim *et al.* described other possible mechanisms by which elevated serum BA levels could affect fetal arrhythmias [57]. Elevated concentrations of secondary BAs are known to cause TGR5-mediated cAMP release in cardiomyocytes without altering contractility. However, secondary BAs act as partial agonists of M2 receptors with a concomitant reduction in contraction rate. Therefore, partial agonism of M2 receptors may serve as a novel mechanism by which BAs induce arrhythmias. This mechanism is expected to be a new target for the treatment of adult and fetal cardiac arrhythmias.

In animal experiments, elevated concentrations of BA tended to lead to arrhythmias and cardiac dysfunction, but UDCA was able to protect the myocardium by antagonizing other hydrophobic Bas [130]. The antiarrhythmic protective effect of UDCA has been validated in a rat fetal cardiac cholestasis model *in vitro* [131]. Another study has shown that UDCA may mediate the antiarrhythmic effect through the increase in cardiac wavelength, which suggested that the treatment of UDCA for arrhythmias has potential value [132]. In a coculture model of neonatal rat CM-myofibroblasts, UDCA can also depolarize myofibroblasts to prevent ventricular conduction slowing and arrhythmias [133]. More recently, data from Ferraro and associates have shown that the effects of UDCA on arrhythmias are not limited to fetal myocardium [134].

UDCA was shown to protect CMs against arrhythmias mediated by adenosine triphosphate-gated K^+^ channels and [Ca^2+^]I [135]. Other studies indicated that UDCA could alter the expression of BA transporters and metabolism-related genes in cardiomyocytes. Hence, it is speculated that the protective effect of UDCA on the heart may be similar to that of dexamethasone in that it has a protective effect on the contractility of cardiomyocytes during arrhythmias.

### 4.5 Bile acid metabolism in heart failure

In patients with chronic heart failure, the serum concentration of secondary BAs was found to have increased, thus resulting in a larger proportion of secondary BAs in the BA pool [136]. BAs act as polar amphiphiles to affect the exchange of sodium and calcium ions on the myocardial cell membrane, inducing backward depolarization of the cell. Subsequent depolarization is one of the initiating mechanisms of heart failure. During the treatment of chronic heart failure, UDCA has been shown to improve peripheral blood flow and liver function in patients by improving vasodilation (both endothelium-dependent and -independent), thereby ensuring NO production in impaired arterial blood flow [137]. In another clinical study, peripheral blood flow improved after extremity ischemia in patients with chronic heart failure who were treated with 500 mg of UDCA twice daily for four weeks [92].

It has been reported that TGR5 agonists enhance the adaptability of the heart to physiological, muscle strength, and hemodynamic stress, further inducing changes in its protective mechanisms [71]. Therefore, TGR5 may be a potential therapeutic target for heart failure. Studies have shown that FXR is downregulated in the left ventricle of spontaneously hypertensive rats with end-stage heart failure [95], but more evidence is needed.

### 4.6 Bile acid metabolism in cirrhosis cardiomyopathy

The deterioration of cholestatic disease leads to liver cirrhosis [138]. The course of patients with cirrhosis is based on the severity of complications caused by changes in the internal structure and overall metabolism of the liver. Patients with visceral and arterial vasodilation develop an abnormal heart rate. Cirrhosis is often accompanied by worsening cardiac output and cardiac insufficiency, as well as changes in cardiac structure and size and impaired function [139, 140]. When the cardiac output increases, arterial blood pressure and systemic vascular resistance decrease, forming a “hyperdynamic circulatory state.” This chronotropic and inotropic cardiac insufficiency is known as “cirrhotic cardiomyopathy,” a type of severe cardiovascular disease characterized by advanced cardiac fibrosis and remodeling [22]. As endogenous amphiphilic products of cholesterol metabolism, BA may be a source of liver cirrhosis and heart disease and is associated with cardiac hypertrophy and atherosclerotic lesions [70].

In models of liver cirrhosis, several mechanisms associated with BAs have been proposed to induce vasodilation of splanchnic and systemic vessels, resulting in hyperdynamic circulation. Therefore, research on the relationship between the pathophysiological characteristics of cirrhosis cardiomyopathy and the abnormal metabolism of BA has sparked interest among researchers [141]. According to previous research results, in the fasting state, the normal level of serum BAs in adults was less than 15 μmol/L, and the serum BA concentration in patients with liver cirrhosis was greater than 100 μmol/L, indicating the development of cirrhotic cardiomyopathy [142]. Therefore, elevated serum BA may be associated with the occurrence and development of cirrhotic cardiomyopathy.

TGR5 can regulate metabolic homeostasis in the heart. Activation of the TGR5 signaling pathway can prevent nonalcoholic steatohepatitis by reducing vascular and liver damage [143]. S1P1R and S1P2R, which are also highly expressed in hepatocytes, are involved in the activation of protein kinase B and extracellular signal-regulated kinase 1/2, regulating vasodilation and increasing blood flow. Therefore, BAs are able to mediate the hemodynamic complications involved in cirrhosis through S1PR. BAs have also been proven to activate the BK pathway and increase the probability of cardiomyopathy in patients with liver cirrhosis [40]. Increased hydrophobic BAs in patients with liver cirrhosis can lead to QT interval prolongation and arrhythmias [144]. BK channels may play an important role in cardiac conduction, and BAs may increase the risk of cirrhotic cardiomyopathy by activating the BK pathway in patients with liver cirrhosis.

In recent years, UDCA, a highly hydrophilic secondary BA, has been shown to act as an alternative drug to protect hepatocytes by promoting bile flow and reducing liver enzyme levels [145]. Furthermore, the replacement of hydrophobic BAs with UDCA reduced cardiac injury in both cirrhotic and noncirrhotic portal stenosis models, suggesting that BAs themselves are important factors in the development of cirrhotic cardiomyopathy [10].

Much of the abovementioned evidence suggests that BAs can affect or regulate the function of the heart in cirrhotic cardiomyopathy. However, to date, there have been few clinical studies specifically targeting the interaction of BAs and cardiovascular function in patients with liver cirrhosis, and more trials are needed in the future.

## 4. Clinical Diagnosis and Application Value of Bile Acids

As the study progressed, researchers have begun to explore the diagnostic and prognostic value, as well as pharmacological application, of BA metabolism and related signaling pathways in cardiovascular and metabolic diseases.

**Figure 3.**
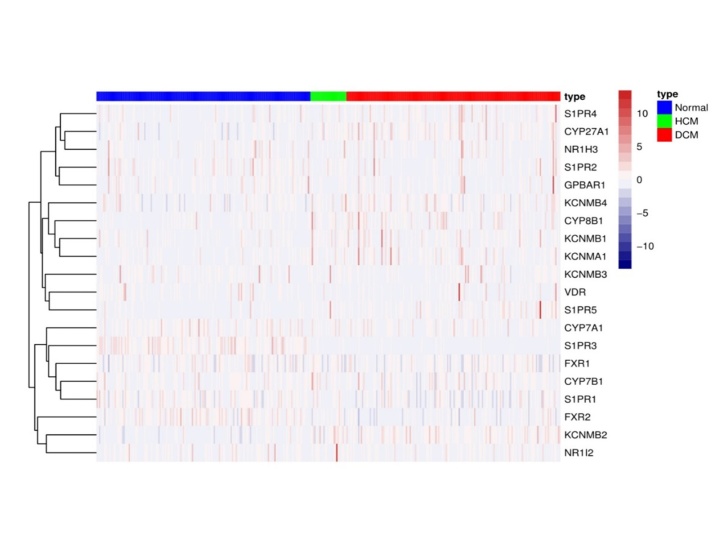
A heatmap analysis of the expression of regulated genes and associated receptors during BA anabolism in DCM and HCM patients based on the accession number GSE141910 from the NCBI GEO database (www.ncbi.nlm.nih.gov/geo).

In a previous study by our team, Liao Y *et al* [146] used metabolomics analysis to explore the changes in systemic and cardiac metabolites in patients with aortic stenosis (AS) before and after transcatheter aortic valve replacement (TAVR) surgery. It was found for the first time that TAVR surgery contributed to a significant increase in primary BA synthesis in patients with AS. Furthermore, based on the accession number GSE141910 from the NCBI GEO database (www.ncbi.nlm.nih.gov/geo), we analyzed the expression level of the regulatory genes and receptors related to BA synthesis and metabolism in dilated cardiomyopathy (DCM) patients, hypertrophic cardiomyopathy (HCM) patients and nonfailing healthy donors. According to the obtained table and heatmap, the expression of most of the BA metabolism-related genes was disturbed compared with that in the nonfailing healthy donors (Table 3, Fig. 3).

In another study, Li W *et al.*[147] investigated the relationship between serum total BAs (TBAs) and coronary artery disease (CAD). Fasting TBA levels were measured in 7,438 participants who underwent coronary angiography, and the results showed that fasting serum TBA levels were positively correlated with the severity of coronary lesions, coronary artery disease, and MI.

As a second-generation bile acid sequestrant (BAS), colesevelam is approved for the treatment of type 2 diabetes mellitus (T2DM) and hyperlipidemia [148]. Colesevelam improves glycemic control in patients with T2DM, but its mechanism underlying the glucose-lowering effect is not fully understood [149, 150]. In clinical trials, colesevelam was able to lower blood glucose levels [151]. Clinical data indicate that colesevelam reduces total plasma cholesterol levels by 10% and LDL-C levels by 15% [152]. Furthermore, colesevelam reduces the risk of CVD by lowering the level of LDL-C in the plasma during the process of cholesterol to bile conversion.

Chevli PA *et al.* [153] investigated the relationship between plasma metabolites and subclinical atherosclerosis in 700 patients with type 2 diabetes and found that coronary artery calcium (CAC) was positively correlated with the BA metabolic subpathways. However, another clinical study initiated by Feng X *et al.* [154] showed that in postmenopausal women with type 2 diabetes, TBA was inversely associated with the occurrence of coronary artery disease and myocardial infarction. This result suggests that sex may influence the association of BA with CVD. Moreover, BAs are inherently sexually dimorphic in humans. Due to the higher activity of 12 α-hydroxylase in women, among the types of BAs, chenodeoxycholic acid levels are higher in women, and cholic acid levels are higher in men [155]. However, the total BA pool in men is larger than that in women [156]. These findings highlight that biological sex is also an important factor to consider when investigating potential treatment strategies.

In a tissue Doppler imaging study, the authors investigated the association of TBA levels and fetal cardiac function in women with intrahepatic cholestasis (ICP). Elevated maternal and fetal serum BA levels in severe ICP have been found to be associated with abnormal fetal cardiac phenotype and fetal cardiac insufficiency compared with those in healthy women with normal TBA levels and in women with mild ICP. In particular, when the maternal TBA level was greater than 440 mmol/L, the incidence of fetal complications, such as spontaneous preterm deliveries, asphyxia events, and meconium staining, was significantly higher [157]. However, the deteriorated fetal cardiac phenotype was partially attenuated by UDCA treatment [158].

In a prospective, single-center study, elevated levels of specific secondary BAs and decreased levels of primary BAs were found in patients with chronic HF. Specifically, Mayerhofer CCK *et al.*[159] measured the plasma levels of primary, secondary and total BAs in 142 chronic heart failure patients and 20 sex- and age-matched healthy controls to explore the association of BAs with clinically relevant variables and the long-term survival rate. It was found that plasma levels of primary BAs decreased, secondary BAs increased and the ratio of secondary BAs to primary BAs increased in HF patients compared with healthy controls. After a median follow-up time of 5.6 years, the patients in the highest tertile (T3) of the ratio of secondary to primary BAs had an approximately twofold mortality rate compared with the patients in the lowest tertile (T1), although this association was weakened after correcting for other confounders. In another single-center study, Voiosu AM et al [160] showed that total BA levels correlated with cardiac output and left atrial volume in patients with cirrhosis. The authors evaluated 58 patients with cirrhosis according to the Child classification, 49 of whom had decompensated cirrhosis. Patients' total BA levels (median, 45 µmol/L) were associated with increased left atrial volume in multivariate analysis and several echocardiographic parameters of hyperdynamic syndrome in univariate analysis.

**Table 3:** Expression of regulatory genes and related receptors during BA synthesis and metabolism in DCM and HCM patients based on the accession number GSE141910 from the NCBI GEO database (www.ncbi.nlm.nih.gov/geo).

Gene symbol	Gene name	DCM	HCM
CYP7A1	/	Down	Down
CYP7B1	/	Satble	Satble
CYP8B1	/	Up	Up
CYP27A1	/	Up	Up
FXR1	/	Satble	Satble
FXR2	/	Down	Down
PXR	NR1I2	Satble	Satble
LXR	NR1H3	Satble	Satble
VDR	/	Down	Down
TGR5	GPBAR1	Satble	Satble
S1PR1	/	Down	Down
S1PR2	/	Down	Satble
S1PR3	/	Down	Down
S1PR4	/	Up	Satble
S1PR5	/	Up	Up
BKCA alpha	KCNMA1	Up	Up
BKCA beta1	KCNMB1	Up	Up
BKCA beta2	KCNMB2	Up	Up
BKCA beta3	KCNMB3	Satble	Satble
BKCA beta4	KCNMB4	Satble	Up

Furthermore, BA homeostasis is jointly maintained by hepatic and intestinal BA signaling pathways. BA induces enterohepatic feedback signals by releasing intestinal hormones and regulates enterohepatic circulation [161]. The role of the gut microbiota as a regulator of intestinal BA metabolism is gradually being implicated in the development of human cardiometabolic diseases by increasing evidence [162, 163]. In addition to BA, trimethylamine nitroxide (TMAO), a gut microbiota-derived metabolite, has recently been implicated in the pathogenesis of CVDs [164, 165]. Both *in vitro* and *in vivo* studies in humans have shown that TMAO has pleiotropic negative effects on the cardiovascular system [166-168]. TMAO promotes atherosclerosis and ventricular remodeling by regulating BA metabolism, leading to vascular dysfunction [169, 170]. Several nonantibiotic small-molecule inhibitors targeting gut microbial choline-TMA lyase are already available [171, 172]. Several preclinical animal model studies have demonstrated that these drugs have great therapeutic potential for various cardiometabolic diseases. They can effectively exert antiatherosclerotic, antiobesity and antithrombotic effects [173, 174]. This also confirms that the gut microbial TMAO pathway is closely related to host BA metabolism and provides another new possible avenue for developing drugs for the treatment of human cardiometabolic diseases.

In addition, in recent years, an increasing number of studies have confirmed the pharmacological applications of bile acid derivatives. BAs are considered to be very helpful for the preparation of novel drugs due to their rigid backbone and potential for surface amphiphilicity [175]. The broad availability, inherent chemical and biological properties and facile derivatization methods of BAs render them useful as scaffolds in drug, supramolecular, and materials chemistry [176].

BAs serve as an attractive cornerstone for designing novel hydrogel systems for the delivery of biomolecules, drugs and vaccines [177, 178]. This has attracted the attention of many researchers, making it a new area of research that warrants increasing attention. BAs may open a new avenue for drug therapy of cardiovascular disease.

## 6. Conclusion and Perspectives

Recently, evidence has accumulated indicating that the relationship between BA metabolism disturbances and CVDs is closely related. When BA metabolism is disordered, a series of cardiac dysfunction and CVDs may also be present. In this paper, we have clarified the metabolic mechanism of BAs and their pharmacological potential in regulating cardiovascular function. BA signaling plays an important role in different cell types through receptor-dependent or channel-mediated mechanisms. Future work should aim to further elucidate the deeper interactions between BAs and their receptors to facilitate the development of new treatments for CVDs.

Current clinical studies as well as our previous metabolomic and bioinformatics analyses have revealed that TBA levels, BA pool composition ratios, and BA-related receptors are partially disturbed in human CVD. However, whether altered BA in humans can serve as a potential biomarker in the pathogenesis of CVD remains unclear and warrants further study.

Fortunately, UDCA has now been found to play a protective role in CVD, although its specific protective mechanism has not been fully elucidated. Currently, some drugs targeting UDCA and its alternatives, some synthetic BA analogs such as OCA and Colesevelam, have been used in clinical practice. However, to further confirm their importance in cardioprotection, more information on their application in preclinical and clinical studies should be provided in the future.

Recent reports have focused on the application of BAs in the preparation of new drugs. BAs have become the main molecules in drug carrier systems due to their good compatibility with different biologically active compounds, showing great potential in medical and biological applications. However, there is no further research on the development of drugs for the treatment of CVDs. Furthermore, the safety and effectiveness of drugs targeting BAs should be evaluated in the treatment of patients with diverse CVDs. Therefore, it is expected that the uniqueness of BAs as drug carriers can be fully utilized to complete further development in the future.

In addition, it is necessary to conduct studies to investigate the effects of drugs that modulate BA metabolism or signaling pathways on lipid metabolism and other related proteins in patients with CVD in the future. This can form a better pharmacological basis for the clinical treatment of CVDs such as atherosclerosis, coronary heart disease and heart failure.

Another potentially interesting area of research is the possible role of VD as a therapeutic target for cardio-vascular diseases. There is evidence that VD may affect cardiovascular function through multiple pathways and that VDR plays an important role in BA transport, metabolism, and detoxification. However, current research on the association between BA and its receptor, VDR, is still insufficient. More research in this area will be needed in the future to further elucidate whether altered VD may serve as a potential biomarker in cardiovascular pathogenesis.

It is worth noting that current research on the mechanism of BA metabolism and CVD progression is mainly carried out in rodents. Nevertheless, their BA metabolism is fundamentally different from that of humans. Some animals do not have gallbladders, such as rats. Therefore, some preclinical findings may vary by species, and the use of animal models to study BA metabolism still has some limitations. Translating findings from animal models into humans is challenging. Future studies need to consider important issues related to species limitations in clinical trial design and seek more efficient ways to explore the potential roles of BA metabolism and BA pool components in human CVD clinically.

Furthermore, the manipulation of factors affecting BA metabolism is unclear. Further research needs to consider whether some factors, including gender dimorphism of BAs and potential signaling crosstalk between gut microbiota and BA signaling pathways, can alter clinical outcomes. Further studies have the potential to open a new era for the application of BA in clinical practice to further prevent the risk of CVD.
